# Bridging the gap: the need for pediatric-specific AI models for tuberculosis detection on chest radiographs

**DOI:** 10.1097/MS9.0000000000004723

**Published:** 2026-01-20

**Authors:** Zainab Farooq, Hamza Sajid, Noor Un Nisa Irshad, Sakan Binte Imran

**Affiliations:** a Department of Internal Medicine, Allama Iqbal Medical College, Lahore, Pakistan; b Department of Internal Medicine, Jinnah Sindh Medical University, Karachi, Pakistan; c Department of Internal Medicine, Sir Salimullah Medical College, Dhaka, Bangladesh

*To the Editor,*

Pediatric TB is a significant global public health issue, with accurate diagnosis being a primary barrier to finding and treating afflicted children. According to WHO Global Tuberculosis Report 2024, an estimated 10.8 million people were diagnosed with TB worldwide in 2023, of which 12% were children and young adolescents. Every year, more than 1 million children under the age of 15 years get tuberculosis; more than half of these cases go undiagnosed and/or unreported because of lack of a sensitive diagnostic test^[1]^. This enormous disease burden emphasizes the difficulty in identifying and reporting pediatric TB cases. Adults, but not children, can be triaged for tuberculosis using computer-aided detection (CAD) systems that automatically read chest X-rays (CXRs). AI is used by these CAD systems to evaluate chest X-ray pictures and identify abnormalities linked to tuberculosis^[2]^.

Due to the availability of sizable labelled datasets, identification of lung abnormalities in adult CXRs by using CA diagnostic algorithms have shown significant success. The number of standard pediatric CXR datasets is small, and the development of pediatric CXR datasets is currently underutilized. This is the biggest barrier to creating and using novel machine learning-based CAD solutions for pediatric CXR in clinical settings^[3]^.

Today, CXRs are still widely used in the clinical diagnosis of pediatric tuberculosis. There are certain particular difficulties with CAD development in children, even if the amount of research on its application in adults is encouraging. Children’s CXR features are more varied and their radiological illness spectrum is broader than those of adults because of their underdeveloped immune systems and other associated conditions. Palmer et al. reported that adult-type tuberculosis primarily affects the lung parenchyma but the hallmark of TB in children is intra thoracic and hilar lymph node enlargement which is mostly represented by narrowing or deviation of the large airways but the current CAD systems show limited ability to identify airway compression and intra-thoracic lymphadenopathy. CAD systems were trained using adult posteroanterior (PA) views, whereas young children are more likely to have anteroposterior (AP) views. This is another factor to take into account when working with a pediatric population^[4]^. Also, axillary lymph node calcification is an occasional consequence of the intradermal BCG vaccination of infants shortly after birth, Trehan *et al* reported in a case report that these calcifications may overlap with radiological signs of tuberculosis and may impair the accuracy of adult trained models^[5]^. Children’s typical physical and anatomical developmental changes across the age spectrum including the existence or absence of the thymus may also be reflected in CXRs, along with the variety of specific abnormalities seen in childhood TB. Pediatric CXR datasets should also ideally represent a wide geographic distribution to account for variations in comorbidities and other respiratory disorders beyond tuberculosis^[6]^. According to multiple studies, there are notable differences in the shape of the lungs from birth to adulthood. When models trained on the adult population are used for lung segmentation in children, this could lead to age-related data domain shifts that would negatively affect lung segmentation performance^[7]^. This implies that before using adult-based AI software to pediatric chest radiographs, more certified data of younger children is required^[8]^.

Recent evaluation of CAD system has shown that its sensitivity for detecting childhood pulmonary TB is suboptimal. These limitations provide strong evidence that there is a need to refine the existing CAD algorithms and to develop pediatric-specific CAD systems that could aid TB detection in children. Based on WHO-standard pediatric CXRs, Chen *et al* created a deep-learning model and reported a high classification performance (AUC = 0.977), indicating that specialized pediatric training can produce accuracy comparable with human readers. These findings emphasize the need for creating extensive and well-characterized libraries of pediatric digital chest radiography images collected from settings with diverse TB epidemiology. This is essential for optimizing the current CAD systems and for developing the much needed pediatric-specific CAD systems in the future which will improve early diagnosis and reduced childhood morbidity^[9,10]^.

This article aligns with the TITAN Guidelines on the need for transparency in AI use in healthcare^[11]^.

## Data Availability

Not applicable.

## Peer and provenance statement

This article was not commissioned and was externally peer-reviewed.
